# 肺癌的下一代测序技术

**DOI:** 10.3779/j.issn.1009-3419.2014.01.10

**Published:** 2014-01-20

**Authors:** Kehua Wu, R Stephanie Huang, Larry House, William Chi Cho, 娟 南

**Affiliations:** 1 Department of Medicine, University of Chicago, Chicago, IL, USA; 2 Department of Clinical Oncology, Queen Elizabeth Hospital, Hong Kong; 3 天津医科大学总医院，天津市肺癌研究所，天津市肺癌转移与肿瘤微环境重点实验室; 4 香港特别行政区 伊利沙伯医院 临床肿瘤科

**Keywords:** 肺癌, 下一代测序, 全外显子组测序, 全基因组测序, 全转录组测序

## Abstract

肺癌在生物学上具有侵袭性，并且是癌症相关死亡的主要原因。根据临床特征、预后、对治疗的反应和耐受性，每一例肺癌患者的进展均是独特的。传统上基于毛细管的单基因测序的第一代技术（如Sanger测序法）已被允许大量平行测序且成本更低、通量更高的下一代测序技术（next-generation sequencing, NGS）所替代。与传统方法相比，NGS技术取得显著进步。我们希望这些方法可全面地解释癌症全球图谱，并提供更多信息以满足个体化用药的需求。本综述包括对不同NGS技术的简要说明，NGS在肺癌研究进展中的应用和重要发现的总结，包括对已知靶基因（*EGFR*、*ALK*和*KRAS*）的进一步探索、其它肺癌突变的鉴定和癌症基因组研究的全局协调。

肺癌在生物学上具有侵袭性，并且是癌症相关死亡的主要原因。全球每年有160万人诊断为肺癌，并有130万人死亡^[1]^。肺癌主要分为两种类型：非小细胞肺癌（non-small-cell lung cancer, NSCLC）（占85%）和小细胞肺癌（约占15%）^[2]^。NSCLC可进一步分为鳞状细胞癌（squamous cell carcinoma, SCC）、腺癌（adenocarcinoma, ADC）和大细胞肺癌。小细胞肺癌的5年生存率仅为6 %，远低于NSCLC的生存率（17%）。伴随手术方法的完善和化放疗的联合应用，肺癌的1年相对生存率从35%（1975年-1979年）升至43%（2003年-2006年）。但是，综合所有分期，生存期超过5年的患者仅有16%。如果肺癌在局限期时即被发现，则5年生存率可达53%。然而，仅15%的肺癌患者在局限期被诊断出来^[101]^。

鉴于肺癌的严重后果及众多治疗失效的经验，研究者将更多的精力投入到认识该病病因学的潜在机制，以期鉴定新型药物靶标。人们很早就认识到，肺癌是一种异质性疾病，而且基因组改变在该病的发生中起至关重要的作用。在过去的几年中，基因组测序技术取得飞速进展。传统上基于毛细管的单基因测序的第一代技术（如Sanger测序法）已被新一代测序技术（next-generation sequencing, NGS）所替代，因为NGS允许大量平行测序，而且具有成本更低和通量更高的特点。与传统方法相比，NGS技术取得显著进步，包括对完整基因组、外显子组和转录组的全面测序。较传统方法，其还具有发现新型染色体重排和拷贝数目改变的优势^[3]^。这些技术进展可革新我们对癌症（如肺癌）的认识。

在本综述中，我们对各种NGS技术做了简要的说明和总结。我们探讨了至今为止NGS在肺癌中的应用及重要发现。但是，NGS在肺癌诊断中的应用不在本综述范围内。

## 下一代测序技术

NGS通常是指第二代和第三代测序技术。这两者的平台比第一代Sanger测序技术均具有更好的能效比和更高的通量。根据目标样本资源和覆盖范围，NGS包括，但不局限于，全基因组测序（whole-genome sequencing, WGS）（针对DNA）、全外显子组测序（whole-exome sequencing, WES）（针对DNA）、全转录组测序（针对RNA的RNA-seq）和靶向靶标测序（DNA和RNA），详细内容如下所述。所有测序的材料预备起始于已剪切的DNA或RNA样本，随后为资料构建。

## 全基因组测序

WGS可以对整个基因组进行测序，提供最全面的基因组特征和最高水平的基因组测序。许多长度为5个-200个核苷酸的随机短DNA片段可以采用此方法同时进行测序^[3]^。人类基因组计划的完成会产生人类基因组序列的总参照，在人类基因组计划完成之前很难解释这些短序列。在所有NGS方法中，WGS花费最高。但是，随着技术的完善，WGS日渐便宜^[3]^。WGS成为一种既可获取全面基因编码序列又有助于明确影响疾病发生和进展因素的有效工具^[4]^。WGS可以检测所有基因组变异，产生了庞大的数据集。数据挖掘仍然是面临的主要挑战之一，必须加以解决，以实现个体化医疗的需求^[4]^。WGS虽费钱费时，但与传统测序方法（Sanger测序）相比，WGS可以识别平衡染色体易位和倒位的断点，从而有了独特的应用^[5]^。此外，WGS可检测基因编码区外发生的基因组变异。这些区域包括非编码的体细胞突变，如启动子、增强子、内含子、非编码RNAs（例如，miRNAs）和未注释的区域^[6, 7]^。激活的遗传变异也可以通过WGS进行鉴定，有助于我们理解遗传性疾病和遗传危险因素引发癌症的本质。WGS可进一步促进疾病的筛查和诊断及个体化治疗的制定^[5]^。

WGS是用于癌症基因组深度测序的主要技术，因为它提供了点突变、融合基因、插入缺失和拷贝数目变异的广泛信息。它还提供了染色体的复杂重排、核苷酸置换突变和重复序列，以及肿瘤的整个基因组的结构重排，包括倒位、易位和复杂重排的信息^[5]^。目前，WGS在癌症基因组研究中最显著的贡献是发现了点突变。在一项WGS研究中，一组配对的正常和肿瘤样本对于区分真正的体细胞突变和遗传性改变的结构重排是必不可少的^[5]^。研究证实全基因组扩增的DNA配对测序对数量有限的细胞是有效的^[8]^。通过这种新颖方法预测的基因组的覆盖范围和深度的测序数据与从未扩增的基因组DNA的预测结果类似。

## 全外显子组测序

WES可用于基因组编码外显子的测序，为发现活化的体细胞突变提供了一种有效方法。传统的基于毛细管的外显子组测序已导致许多疾病中靶向体细胞突变的重要发现。就肺癌而言，部分突变发生于EGFR和ALK受体酪氨酸激酶^[3]^。尽管WES局限于有注释基因的变异的编码和拼接位点，但是它还可以检测整个基因组中已知编码基因的突变，具有较高的覆盖性。WES不能检测启动子或调节区的改变^[9]^。起初，WES只用于外显子组。目前它已扩展至基因组的多个区段，包括外显子侧翼区、启动子、非翻译区和miRNA基因的非编码DNA。单个样本的WES检测发现了多达25, 000个变异，导致鉴定活化突变的挑战。WES和WGS的最大区别是产生数据的数量和内容的不同。一般而言，如果旨在发现目标突变，WES更合适。WES用户可受益于每个样本的花费和分析时间的减少，感兴趣区域的覆盖范围的增加以及突变信息的准确性的提高^[10]^。事实上，干扰蛋白功能的基因编码区域的突变或变异可被WES识别，而结构和非编码变异仅能被WGS检测。请注意，许多用于WGS的平台还适用于WES。一项重要挑战是从伴随变异中识别致病突变，而伴随变体与疾病病因无关^[11]^。WGS和WES在检测插入缺失、三核苷酸重复和拷贝数目的变异中均有困难^[9]^。

WES为罕见的孟德尔遗传疾病的致病基因组变异的发现提供了难得机会^[12-15]^。WES主要应用于表征单基因疾病（孟德尔遗传疾病）的缺陷，这些缺陷可引起罕见的家族性疾病。从小型家族收集的个别罕见基因变体突变可被WES识别。这种方法亦可用于发现活化的突变。外显子突变或拼接位点突变是多数孟德尔遗传疾病的主要原因^[12]^。此外，外显子组测序可能在非遗传性或新生突变相关疾病中也具有较大潜能^[15]^。比如，它可能促进肿瘤样本中已知突变的诊断和新型突变的鉴定。

## RNA测序

对从细胞中提取的RNA的测序（sequencing of RNA extracted from cells, RNA-seq）通过读取短cDNA序列片段和基于参照基因组进行绘制，产生了完整的转录组的全部序列。RNA-seq使得绘制外显子和内含子的边界、以及基因的5´和3´末端成为可能，并导致人们对转录组的复杂性有了更全面的了解^[16]^。传统意义上，RNA-seq要求在资料构建前通过杂交对poly(A)进行选择，以便富集RNA聚合酶Ⅱ转录物和/或减少核糖体RNA的含量，该领域的进展使得我们直接可以在总RNA量为皮克时进行工作，无需分馏步骤，分馏步骤会限制转录组的完整测序。RNA-seq在检测框内融合^[3]^、体细胞突变^[3]^和基因表达序列分析^[5]^时具有敏感性和有效性。在量化丰富的转录物，尤其是当这些转录物表达较低时，RNA-seq较微阵技术具有更高的敏感性^[17]^。RNA-seq技术不仅可用于分析既往已知基因，而且可检测当前未知转录物、选择性剪接物和非人类转录物^[5]^。RNA-seq面临三大挑战：资料构建、数据挖掘和覆盖范围与花费的比较^[18]^。

## 靶向测序

靶向测序通过只对靶区域测序而仅用于感兴趣的基因组区域，以期达到节约时间和成本效益的分析。靶基因通常限定于既往已知癌症相关的变异。与WGS相比，靶向测序产生了更小的数据库，因此需要更简便的数据分析。该方法可能漏掉既往未知的突变。

## NGS在肺癌中的应用

自2008年NGS在肺癌中首次应用发表后，多项研究采用不同的NGS方法研究除肺癌外的不同疾病。表 1总结了这些研究的疾病类型、临床样本、所采用的NGS方法、范围、所用平台和主要发现。其它细节如下所述。

**1:** 下一代测序在肺癌中的应用总结

研究(年）	患者（*n*）	NGS方法	样本	平均（或中位）测序深度	NGS平台	主要发现	参考文献
Marchetti等 (2012）	NSCLC Ⅲ期-Ⅳ期患者（116例）	ES	DNA	-	454 GS Junior Technology^†^ (Roche)	*EGFR*外显子19的复杂突变和缺失亚群	[28]
Su等 (2012）	NSCLC患者（192例；107例未采用TKI治疗和85例采用TKI治疗）	-	DNA	-	MALDI-TOF MS MassArray® System (Sequenom)	在采用EGFR TKIs治疗的患者中，对T790M进行预处理是PFS缩短的预测因子	[30]
Jung等 (2012）	NSCLC细胞系（2种）	RNA-seq	RNA	750, 000个序列，其中一个序列为400 bp	454 GS FLX Instrument (Roche)	*ALK-PTPN3*基因融合	[31]
Kalari等 (2012）	肺ADCs（15例；8例携带*KRAS*突变，7例无*KRAS*突变）	RNA-seq	RNA	-	Agilent Bioanalyzer (Agilent Technologies)	374个不同的基因表达，259个基因交替拼接和65个SNV相关改变；3个受影响的通路：NF-xB、ERK1/2和AKT	[32]
Ju等 (2012）	肺ADC；从不吸烟者（1例）	WGS, RNA-seq	DNA, RNA	47.77×和28.27×	Illumina HiSeq^TM^ 2000 and Genome Analyzer Ilx (Illumina)	*KIF5B*和*RET*基因融合	[33]
Kohno等 (2012）	肺ADCs（433例）	RNA-seq	RNA	229×	Genome Analyzer Ilx (Illumina)	*KIF5B*和*RET*基因融合	[34]
Lipson等 (2012）	NSCLCs Ⅰ期-Ⅳ期（40例）	WES	DNA	229×	Illumina HiSeq 2000 instrument (Illumina)	*KIF5B*和*RET*基因融合	[35]
Imielinski等 (2012）	肺ADCs Ⅰ期-Ⅳ期（183例）	WGS, WES	DNA	92× (WES)和69× (WGS)	Illumina HiSeq Platform (Illumina)	*U2AF1*的体细胞突变，与*RBM10*和*ARID1A*相关的截断突变；*EGFR*和*SIK2*激酶的基因变异	[36]
Ding等 (2012）	肺ADCs（188例）	WGS	DNA	-	各种平台	26个突变基因（如*TP53*、*CDKN2A*、*STK11*、*KRAS*、*EGFR*、*NRAS*、*NF1*、*ATM*、*RB1*、*APC*、*LRP18*、*PTPRD*、*ERBB4*、*ERBB3*、*ERBB2*和*EPHA3*）	[37]
Greulich等 (2012）	肺ADCs（188例）	WGS	DNA	-	各种平台	*ERBB2*的胞外区突变和*ERBB2*突变的激活机制	[38]
Liu等 (2012）	NSCLC患者（16例；1例肺ADC，12例肺SCCs，1例肺腺鳞癌，1例BAC和1例肺ADC含BAC）	WES	DNA	123×	Illumina Genome Analyzer Ilx和HiSeq 2000 (lllumina)	发现突变频次居第二位的新基因*CSMD3*；*CSMD3*缺失会导致气道上皮细胞增生增多	[45]
Pleasance等 (2010）	1种SCLC细胞系（NCI-H209），源自1例55岁、男性、未化疗、吸烟史未知的SCLC患者	WGS	DNA	肿瘤组织39 ×；正常组织31×	SOLiD platform	22, 910个体细胞代替（134个位于编码外显子区）；转录偶联和表达相关的DNA修复途径；烟草中的致癌物；*CHD7*可能反复地重排	[47]
Keller等 (2011）	NSCLC患者（10例）	RNA-seq	RNA	-	SOLiD 4 analyzer	76个新miRNAs和41个已知前体细胞的成熟形式；32个带注解的和7个未知的miRNAs	[48]
Lee等 (2010）	51岁男性吸烟白种NSCLC（1例）	WGS	DNA	肿瘤组织60 ×；正常组织46×	-	530个体细胞单核苷酸变异，其中一个位于*KRAS*，391个位于编码区，43个大规模结构变异，激酶基因中的氨基酸改变的突变更高	[49]
Carvalho等 (2012）	NSCLC患者（48例）	MethyICap-seq	DNA	-	Illumina Genome Analyzer Ilx (Illumina)	ADCs和SCCs中57个明确的DMRs；一些DMRs为与编码转录调节因子的基因相关的甲基化	[50]
Beane等 (2011）	3个健康的从不吸烟者，3个健康的吸烟者；8个吸烟的肺癌者；5个吸烟但肺癌症者（19例）	RNA-seq	RNA	-	Illumina Genome Analyzer Ilx (Illumina)	在吸烟的肺癌患者中，发现趋化因子信号通路，细胞因子-细胞因子受体的相互作用和细胞粘附分子差异表达；一个内含子中的*MUCSAC*转录物下调	[51]
^†^454 GS Junior system为一种基于焦磷酸测序的方法。 ADC：腺癌；BAC：细支气管肺泡癌；DMR：不同的甲基化区域；ES：外显子组测序；MALDI-TOF：基质辅助激光解吸电离飞行时间；MS：质谱；NGS：下一代测序；NSCLC：非小细胞肺癌；PFS：无进展生存期；RNA-seq：全转录组测序；SCC：鳞状细胞癌；SCLC：小细胞肺癌；SNV：单核苷酸变异；SOLiD：基于寡核苷酸连接和检测进行测序；TKI：酪氨酸激酶抑制剂；WES：全外显子组测序；WGS：全基因组测序。 注：本表得到版权所有者© 2013 Future Medicine Ltd复制许可。							

## NGS对既往已知靶基因的其它发现

### EGFR

在癌症，尤其是NSCLC中，EGFR通过对可调节增殖和凋亡的信号转导通路进行调控，在肿瘤发生中起关键作用。EGFR的突变频率见表 2 ^[20-26]^。*EGFR*为NSCLC的重要基因变异，靶向EGFR的酪氨酸激酶抑制剂（tyrosine kinase inhibitors，TKIs；如吉非替尼和厄洛替尼）的出现为改善NSCLC的治疗点燃希望。但是，由于其在一般NSCLC患者群中缺乏疗效，最初的热情受到挫伤。随后，研究显示*EGFR*突变对EGFR TKIs的疗效至关重要^[20]^。2011年，美国临床肿瘤学会发布了一项初步临床意见指出，采用EGFR抑制剂进行一线治疗需基于阳性突变^[27]^。在过去的十年中，对可预测肺癌患者对EGFR TKIs的疗效的生物标记物的进一步研究是一个活跃的研究领域。NGS技术在与EGFR TKI敏感性相关的其它基因标记物的发现中起重要作用。

**2:** *EGFR*、*ELM4-ALK*、*KRAS*和*BRAF*的突变频率

患者	*EGFR* (%)	*ELM4-ALK* (%)	*KRAS* (%)	*BRAF* (%)
非吸烟的美国人	19-27	50	52	4
吸烟的美国人	4-7	29	34	1
非吸烟的亚洲人	49-76.2	4.3-11	2-5	1.92^†^
吸烟的亚洲人	43.5	3	16.5	3
^†^东亚女性腺癌。 注：本表得到版权所有者© 2013 Future Medicine Ltd复制许可。				

主要的*EGFR*突变为外显子19的框内缺失，这是采用EGFR抑制剂进行个体化治疗的有效标记物。Marchetti等对116个NSCLC的DNA样本通过传统Sanger和NGS的测序结果进行了比较^[28]^。其中，有106个样本采用Sanger测序显示外显子19缺失，采用NGS分析也可见此突变。但是，仅88%（93个）的样本通过NGS方法验证与Sanger测序具有相同的缺失。有13个（12%）病例的缺失通过两种方法测序的特征不同。在这些缺失中，有6个样本的缺失起点/终点未被Sanger测序检测出且无法合理解读。其它7个病例的缺失起点/终点明确，虽然缺失基质的恰当序列未被Sanger测序定义（如c.2240_2254del和c.2239_2262del），但可被NGS定义。通过NGS测序，在21个样本（20%）中发现双倍或多倍框移缺失相关的框内改变。通过Sanger测序，16个病例的缺失为长的和短的非框内缺失（17 bp_del和1 bp_del），被认定为缺失和插入。其它5个样本被发现携带复杂缺失，这些缺失有可能是新的突变。这些突变包括：c.2239_2248del c.2253_2260del、c.2230_2237del c.2245_2251del、c.2239_ 2262del c.2263_2274dup（插入）、c.2236_ 2252del c.2258del和c.2234_ 2236del c.2241_2252del。除了主要缺失，还有携带不同缺失的DNA分子亚群，它们在结构上与主要缺失有关。NGS检测46个（43%）样本发现，每一亚群占有肿瘤基因组的0.1%-17%。外显子19的复杂突变和缺失亚群的发现有可能解释携带外显子19突变的NSCLC患者的不同反应[例如，反应率：53.3%-75%；无进展生存期（progression-free survival, PFS）：7.1个月-398天] ^[20]^。这项研究还说明，外显子19的某一区域也是高度变化的，这可能影响EGFR TKIs的个体化治疗和肺癌的发病机理^[28]^。NGS有可能更全面地绘制EGFR外显子19的缺失序列，由于该缺失的不同形式可能导致不同EGFR TKI反应、疾病进展，并可影响携带外显子19缺失蛋白的抗原性^[28]^。

除了外显子19的缺失，外显子20的T790M也显著影响着患者对EGFR TKI治疗的反应。该点突变主要与EGFR TKI治疗的获得性耐药相关。比如，在肺ADC中，T790M突变导致50%以上的获得性耐药^[25]^。研究显示，在TKI治疗后可获得*EGFR* T790M突变^[29]^。此外，EGFR T790M突变预处理在预测EGFR TKIs疗效中的作用被研究。Su等采用直接测序、基质辅助激光解吸电离飞行时间（Matrix-assisted laser desorption/ionization-time of flight, MALDI-TOF）质谱分析和NGS方法分析了EGFR TKIs治疗前后的NSCLC患者的DNA样本^[30]^。这些患者混杂着吸烟者（23.4%）和非吸烟者（76.6%），ADC（94.3%）或SCC（5.7%）。研究发现预处理和未处理T790M的患者的PFS间有统计学差异（*P*=0.0298），而不同基因型的组别间对EGFR TKIs的反应率和总生存期无明显差异。仍有57%的携带T790M突变的患者对治疗是有反应的，该现象尚不能解释。EGFR TKIs治疗使T790M突变从治疗前的31.5%升至治疗后的83.3%。

### ALK

最近，作为新兴生物标记物和治疗靶标的ALK酪氨酸激酶受体受到了广泛关注。*EML4-ALK*易位是最常见的*ALK*基因重排。大约2%-11%的肺癌患者的*EML4-ALK*为阳性^[20]^。采用基因组DNA测序发现1例43岁非吸烟男性NSCLC患者含有复杂的*ALK*重排，但根据Vysis ALK Break Apart FISH分析（Abbott Molecular Inc., IL, USA）其未携带突变的*EGFR*和*EML4-ALK*重排。连同其它的cDNA测序，研究显示复杂重排包括至少5个不同的基因座内有断点。其中之一为*ALK*内含子19和典型的*EML4-ALK*融合（*EML4*外显子1-13，*ALK*外显子20-29）。与外显子1-19相比，外显子20-29的表达高至39倍。此类患者在服用克唑替尼后疾病有改善。由于NGS可以检测复杂的*ML4-ALK*重排，而传统的FISH分析不能检测这些重排，所以可以考虑采用NGS来为NSCLC患者制定治疗方案。

Jung和同事们对NSCLC组成的cDNA样本进行转录组测序。通过NGS发现了228个融合转录物候选因子^[31]^。排除可能的假阳性后降至16个。大多数融合基因里均有一个单序列。通过逆转录-PCR进一步证实了候选融合基因，而且仅从H2228细胞中产生了一个成功扩增的融合基因（*PTPN3-ALK*）。5’-RACE产物分析显示，*ALK* 5’的第11个内含子至第8个内含子片段转位至*PTPN3* 5’端的第2个外显子和第1个外显子。此融合基因导致*PTPN3*一个等位基因的失活，*PTPN3*具有肿瘤抑制活性，并且是非受体型蛋白酪氨酸磷酸酶的成员。这些发现有助于肺癌的诊断和治疗。

### KRAS

众所周知，携带*KRAS*突变的NSCLC具有侵袭性，并且对EGFR TKIs耐药。研发靶向突变*KRAS*的治疗方法较为困难。通过Agilent Bioanalyzer（Agilent Technologies Inc., CA, USA）对15个原发肺癌肿瘤样本进行双端RNA测序来识别携带*KRAS*突变的患者中明确改变的通路。人类基因组和基因组图谱被描述。网络分析显示在*KRAS*突变的肿瘤中存在差异表达（374个基因）、选择性剪接（259个基因）和单核苷酸变异（single-nucleotide variant, SNV）相关的改变（65个基因）。在该项研究中，NFkB、ERK1/2和AKT通路被证实可以激活，还发现了突变*KRAS*、TNFR和PPAR_γ_信号通路的关系。这些发现对相应的靶向治疗更为敏感，这将为EGFR TKI-耐药的NSCLC患者提供替代的治疗选择^[32]^。

## 探索性研究中其它肺癌突变的鉴定

### *KIF5B*和*RET*融合基因

最近有3项研究报道了肺ADC患者中存在*KIF5B*和*RET*基因融合的新转化^[33-35]^。在日本和美国的肺ADC患者中，该新型融合的发生率为1%-2% ^[34]^。*KIF5B*和*RET*融合基因仅见于肺ADC患者，而不是肺癌的其它类型（SCC、大细胞或小细胞肺癌）或ADC的其它类型（卵巢和结肠）^[33-35]^。在许多肿瘤中*RET*是已知的致癌基因，如胰腺癌和前列腺癌。融合伙伴*KIF5B*有一个可激活致癌基因的卷曲螺旋域。该融合基因可以引起RET激酶的异常活化，并同时诱导细胞转化^[33, 34]^。它是一个额外的新型驱动突变，因为它是在3项独立的研究中被发现的，且与既往已知的肺ADC突变或融合基因（如*EGFR*、*KRAS*和*EML4-ALK*）互相排斥^[33-35]^。*KIF5B*-*RET*融合基因是个体化靶向治疗的潜在靶标，而且可以通过多种靶向激酶抑制剂来成药，如舒尼替尼、索拉菲尼以及刚被批准的凡德他尼^[34, 35]^。Ju等通过抑制RET的磷酸化，报道了对1例肺ADC患者的癌组织和配对正常组织的大规模并行全基因组和转录组测序的整合分析^[33]^。此患者为33岁男性、非吸烟且无家族癌症史。他未携带突变的*EGFR*、*KRAS*或*EML4-ALK*。血液DNA的WGS分析显示，他的癌症并非种系突变所驱动，因为他不含有任何SNPedia[单核苷酸多态性（single-nucleotide polymorphism, SNP）数据库]中已归档的明显与癌症相关的SNVs。通过比较肝脏中的转移癌和正常组织，识别了10个非同义体细胞突变（8个SNVs和2个插入缺失）。这10个突变并不位于已知的靶基因（*EGFR*、*KRAS*和*BRAF*）上，其并非驱动突变，甚至在原发肺转移癌中也不是。通过转录组测序对融合基因的进一步分析发现了52个融合基因。其中，49个（94.2%）为相邻基因的染色体内融合，1个由于触珠蛋白而产生。这503个基因在肿瘤发生中不起功能性作用。其它2个融合基因为*KIF5B*-*RET*和*KIAA1462*-*KIF5B*。它们为染色体内疏远基因（超过~2 Mb）的融合。鉴于*KIAA1462*-*KIF5B*表达较低，且KIAA1462为分子功能未知的假定蛋白，故不对其进行进一步地分析。所有52个融合基因中，在肝转移性肺组织中通过WGS仅在*KIF5B*-*RET*中检测到染色体重排（如大的缺失、倒位或易位）。RNA-seq发现此融合基因高表达34个不一致的双端序列和60个跨越融合交界的生长序列。分析还发现，*KIF5B*的第16个外显子末端与*RET*原癌基因的第12个外显子的起始端整合。10p11.22-q11.21的臂间倒位导致融合基因*KIF5B*-*RET*。这些外显子的RNA表达水平显示，大部分*RET*表达来自融合基因，而不是野生型*RET*基因。随后的重复研究在20个原发肺ADC的2个病例中证实了*KIF5B*-*RET*融合基因的出现。*KIF5B*中DNA双链断裂似乎是可变，3个患者中*RET*的第12个外显子与*KIF5B*在不同位点结合（所检测的患者为外显子16，2个验证的患者为外显子15和23）。研究显示，*KIF5B*-*RET*融合基因为NSCLCs的一个子集，还指出嵌合癌基因是采用相应抑制剂进行未来个体化治疗的潜在分子靶标。

最近，有2项研究还发现肺癌中存在*KIF5B*-*RET*融合^[34, 35]^。Kohno等采用逆转录PCR和Sanger测序分析了319个日本ADC患者的样本^[34]^。其中，30个采用RNA-seq进行分析。研究发现6个非吸烟患者携带*KIF5B*和*RET*融合基因。资料显示，染色体10p11.2上的*KIF5B*内含子15、16、23或24与染色体10q11.2上的RET内含子7或11融合。FISH验证了染色体10的着丝粒区域的长臂和断臂间存在重排。*KIF5B*-*RET*融合阳性患者的RET表达比其他人高30倍，与Ju等的结论一致，其结论认为大部分*RET*癌基因来自融合基因^[33]^。在另一项81例美国患者和34例挪威患者的研究中，1例曾吸烟的美国患者存在*KIF5B*-*RET*融合阳性。*KIF5B*-*RET*融合和吸烟状态的关系未明。在融合阳性的患者中发现*KIF5B*-*RET*蛋白Tyr905的磷酸化，提示*RET*融合是既往未知的突变。在另一项Lipson及其同事的研究中，研究者分析了24个福尔马林固定石蜡包埋的NSCLC组织样本^[35]^。发现1例44岁男性非吸烟者存在*KIF5B*-*RET*融合阳性。进一步的筛查发现，11例患者（561例肺ADC患者中）存在*KIF5B*-*RET*融合。

### 其它新发现

“癌症标志”是指一组对肿瘤发生至关重要的细胞特性。Imielinski等通过对183例肺ADC肿瘤和正常DNA配对的全外显子组或全基因组进行大规模并行测序绘制了癌症标志^[36]^。这183个病例的外显子区域存在77, 736个体细胞变异，平均为11.9个突变/Mb。与非吸烟患者相比，吸烟患者的外显子突变率明显升高（*P*=1.9×10^-9^）。CpG二核苷酸的C→T转换（CpG→T）和C→A颠换是最常见的突变特征。A→C最少见。5个突变谱聚类与患者的临床特征相关。聚类1（富集CpG→T突变，且总突变率较低）多见于非吸烟或轻度吸烟的患者（*P*=3.0×10^-9^）。聚类4（除了CpG结构和TpC突变为T或G外，还有C→ T）与晚期明显相关（Ⅲb期或Ⅳ期；*P*=0.006, 3）。4个高突变率的分析发现了共计25个明显的突变基因，部分基因之前已知，为*TP53*（频率与既往报道一致，为50%）、*KRAS*（27%）、*EGFR*（17%）、*STK11*（15%）、*KEAP1*（12%）、*ATM*、*NF1*（11%）、*BRAF*（8%）和*SMAD4*（3%）。其余未见报道，为*SMARCA4*、*ARID1A*、*RBM10*、*SETD2*、*PICK3CA*、*CBL*、*FBXW7*、*PPP2R1A*、*RB1*、*CTNNB1*、*U2AF1*、*KIAA0427*、*PTEN*、*BRD3*、*FGFR3*和*GOPC*。对这25个基因的进一步相关分析显示，*EGFR*突变与*KRAS*突变明显相斥（*P*=3.3×10^-4^）；*EGFR*突变与非/轻度吸烟（*P*=2.0×10^-6^）及突变谱聚类1相关（*P*=0.001, 5），*KRAS*、*STK11*、*SMARCA4*和*KEAP1*与突变谱聚类1和非/轻度吸烟状态负相关（*P* < 0.005）；突变谱聚类3除了CpG外还有C→A颠换，富含*KRAS*变异（*P*=0.000, 71）；*STK11*与突变谱聚类2（CpG→T转换和CpG→A颠换；*P*=0.002, 6）相关；*NF1*与*U2AF1*可同时发生。PFS的相关分析显示，*U2AF1*和*TP53*突变导致生存期缩短（分别为*P*=0.000, 11和0.001, 4）。*U2AF1*为最明显的突变基因之一，在患者中的发生率为3%。5个病例中有4个发现相同的c.101C>T、p.S34F突变。在这4个病例中，1例为KRAS突变阳性，提示*U2AF1*具有独立地致癌作用。*RBM10*占突变的7%（12/183）。*RBM10*高表达，为RNA结合蛋白。*RBM10*与*KRAS*、*EGFR*或*PIK3CA*可同时发生。*ARID1A*在患者中的阳性率为8%，在SWI-SNF染色质重塑复合物中编码重要的蛋白质。WGS揭示了范围广泛的总重排、基因重排和基因组的复杂性。通过检测肺癌中新的激酶基因的潜在活化框内重排发现了*EGFR*的两个外显子缺失。这一重排发生在*EGFR*的C末端（由外显子25和26编码），且可见另一变异（p.G719S突变）。在NIH 3T3细胞中进一步研究了该突变，发现EGFR和AKT磷酸化增多，EGF磷酸化未见增加。在Ba/F3细胞中，致癌*EGFR*缺失突变导致增殖，该增殖可被厄洛替尼（一种EGFR TKI）阻止。WGS分析还发现*SIK2*和*ROCK1*的框内重排。该研究有助于肺ADC基因组变异的完成和表征。

Ding等报道了一项在188例肺ADC中进行的合作研究，发现了26个与癌变相关的高变频基因，包括*EGFR*同类物*ERBB4*、*ERBB3*和*ERBB2*^[37]^。在这26个基因中，Greulich等选择了6个受体酪氨酸激酶基因（*EPHA3*、*ERBB4*、*FGFR4*、*NTRK3*、*NTRK2*和*ERBB2*）来研究肺ADC中基因突变的功能意义^[38]^。他们发现*ERBB2*的致癌胞外区突变，该突变可使NIH 3T3细胞具有锚着独立性。ERBB2的活化通过增加羧基端尾部的磷酸化或共价二聚化来启动。胞外区突变的ERBB2的抑制剂为治疗选择提供了希望，促使了一项临床试验来研究肺癌中ERBB2的抑制剂。他们的结果与癌症基因组图谱（The Cancer Genome Atlas, TCGA）的结论一致，TCGA结论认为ERBBs可能是以下讨论的一种分子靶标^[39]^。

## 癌症基因组研究的全局协调

国际癌症基因组协会（International Cancer Genome Consortium, ICGC）举国际之力调查癌症的全球图谱并全面鉴定与肿瘤相关的基因组、转录组和表观基因改变，包括50种癌症类型的基因组缺失或扩增、体细胞突变、基因重排、表观遗传修饰和基因的异常表达^[10, 102]^。目前，15个行政辖区的51个组已加入ICGC，包括亚洲、澳洲、欧洲和北美洲，共研究超过24, 000个肿瘤基因组^[102]^。TCGA计划于2005年由国家癌症研究院和国家人类基因组研究院启动，有助于ICGC。TCGA通过NGS技术为癌症分子特征提供了更全面的认识。TCGA纳入了超过20种癌症常见类型（包括肺癌），每种癌症类型有500个样本。致力于不同但相关项目的研究和技术团队的国家网络表现出了极大的进展，总结见表 3^[40, 103]^。

**3:** 癌症基因组图谱项目公布的肺癌的下一代基因测序

研究（年）	患者（*n*）	NGS方法	样本	平均（或中位）测序深度	NGS平台	主要发现	参考文献
癌症基因组 图谱研究网络 （2012）	肺SCCs；96%有吸烟史（178例）	WGS; WES; RNA-seq	DNA; RNA	51×(WGS) 121×(WES) 19×(RNA-seq)	Illumina HiSeq^TM^ (Illumina)	360个外显子突变, 165个基因组重排；323个拷贝数目变异，10个复发突变基因，4条明显变异的通路，3个潜在的分子靶向策略	[39]
Govindan等 （2012）	肺SCCs；96%有吸烟史（178例）	WGS; WES; RNA-seq	DNA; RNA	-	-	通过mRNA表达谱发现30个明显的体细胞拷贝数目变异，13个突变基因，4个表达亚型（经典的、基础的、分泌型的和原始的）；72%病例可见*CDKN2A*缺失，75%的病例具有采用已有可用药物的潜在分子靶标	[41]
NGS：下一代测序；RNA-seq：全转录组测序；SCC：鳞状细胞癌；WES：全外显子组测序；WGS：全基因组测序。 注：本表得到版权所有者© 2013 Future Medicine Ltd复制许可。							

作为TCGA项目的一部分，一项旨在全面表征SCC基因组和表观基因组图谱、发现SCC潜在治疗方法的大型队列研究于近年公布^[39]^。在该研究纳入的78例患者中，96%有吸烟史。研究发现了复杂的基因组变异，每一肿瘤平均含有360个外显子突变、65个基因组重排和323个拷贝数目变异的片段。染色体3q的选择性扩增是SCC与肺ADC的最大区别。研究发现有50个峰存在明显的扩增或缺失。在这些变异中，有一些体细胞拷贝数目变异曾被报道，包括*SOX2*、*PDGFRA*和/或*KIT*、*EGFR*、*FGFR1*和/或*WHSC1L1*、*CCND1*和*CDKN2A*。对SCC患者而言，最常见的突变为CpG的转换和颠换。该研究识别出0个复发突变基因（*TP53*、*CDKN2A*、*PTEN*、*PIK3CA*、*KEAP1*、*MLL2*、*HLA-A*、*NFE2L2*、*NOTCH1*和*RB1*）。*TP53*突变最为频繁（81%）。有研究识别出4条明显变异的通路，为*NFE2L2*和*KEAP1*和/或*CUL3*缺失或突变（34%），鳞状细胞分化（44%），磷脂酰肌醇-3-OH激酶通路（47%）以及CDKN2A和RB（72%）。*KEAP1*和*CUL3*突变与功能丧失相关。鳞状细胞分化基因包括*SOX2*和*TP63*的过表达和扩增，*NOTCH1*、*NOTCH2*、*ASCL4*的功能丧失突变和*FOXP1*的局部缺失。*CDKN2A*是编码p6INK4A和p14ART蛋白的肿瘤抑制基因。

伴随潜在靶标基因的筛查及药物研发的需要，研究者建议了3个分子靶向策略：*ERBBs*、*FGFRs*和*JAKs*，这三者均被发现有突变和/或扩增。*ERBB2*的结果与Greulich等的结论（*ERBB2*可能是靶向治疗的独特机会）^[38]^相符。此外，Govindan等在202年的美国临床肿瘤学会会议上呈现了78例SCC患者的全部基因组特征^[4]^。他们发现了30个明显体细胞拷贝数目变异的位点和3个明显突变的基因（包括*TP53*、*CDKN2A*、*PTEN*、*KEAP1*和*NFE2L2*），识别出4个不同的表达：*NFE2L2*和*KEAP1*突变、FGFR激酶变异、总体甲基化增加和烟草使用率最高。72%的病例存在*CDKN2A*缺失。对于现有可用药物，75%的患者含有潜在的分子靶标。

TCGA整合了研究机构间大量系统网络的测序数据。事实上整个研究机构可以访问所有的数据资料，从而加速从研究发现到临床的转化工作。TCGA组的进展显示许多基因组信息穿插在不同的肿瘤类型间^[42-44]^，包括肺癌。此外，源自TCGA的数据也可能用于验证原创研究的结果^[45]^，或作为质量控制^[46]^。

## 未来前景

2003年人类基因组计划完成后，NGS技术改进明显增加了基因组数据量，这些数据由检测罕见结构变异中基因组覆盖范围和细微差别增多的研究产生。随着技术改进，这些技术在研究中的实用性及其潜在临床应用也有所增加。NGS方法允许我们深入挖掘遗传密码，以识别大量潜在的基因和基因组差异（如结构异常、拷贝数目增加和体细胞SNVs）。他们为我们提供了解密癌症基因组中复杂基因改变的工具。通过提供既往已知疾病/治疗相关基因靶标的全面基因组图谱，NGS可能提高我们选择恰当的治疗药物的能力。而且，NGS发现了许多其它的基因组标记物，它们对致病/治疗很重要。通过该方法产生的数据使研究者可以实施旨在提高我们对生物学和患者治疗的认识的系统生物学研究，最终实现真正地个体化用药。如本综述所总结，NGS为探索更多的基因变异提供了机会，这些基因变异可能导致有前景的治疗靶标。此外，国际间合作网络通过研究机构分享数据库和技术，如ICGC，会加快每一个体患者突变图谱发现的步伐。NGS相关的临床挑战是，所述病症并不总是与临床实践中所观察到的疾病相关^[5]^。通过使用有效的靶向治疗，大多数现已识别的突变并未转化至临床实践。点突变的有效使用仍为实现癌症个体化用药的一项挑战。

即使有了这些进展，挖掘NGS分析产生的庞大数据集进行分析并将其转化为医疗保健仍具有挑战性。疾病的异质性以及患者伴有其它环境影响，增加了解释NGS结果的难度。为了将NGS结果真正地转化为临床癌症治疗，需要进一步采用先进的软件程序进行生物信息学分析、对NGS结果进行功能验证和临床试验确认。

有证据表明，携带既往已知靶标如*EGFR*和*ALK*重排的患者可获益于相应的抑制剂治疗^[20]^。传统方法无法解释一些患者可获益于临床上地初始治疗，但会发展为耐药并对该治疗产生不同反应。NGS可能通过采用更多的全面测序来明确病因。比如，Marchetti等发现了外显子9的复杂突变和缺失亚群^[28]^。NGS有助于为患者寻找恰当的靶向治疗。而且，现有可用的分子靶向药物在肺ADC中普遍使用。NGS还有望发现其它肺癌类型的药物靶标。此外，NGS无疑会改善癌症诊断，这些不在本综述范围之内。预计将有更有效的靶标和生物标记物被发现和验证，就像采用NGS分析所产生的癌症领域的发现会有持续进展一样。NGS技术还有助于提高我们对癌症致病基因突变/变异的认识，期待有更多生物标记物和潜在药物靶标通过NGS被发现。

## Financial & competing interests disclosure

The authors have no relevant affiliations or financial involvement with any organization or entity with a financial interest in or financial conflict with the subject matter or materials discussed in the manuscript. This includes employment, consultancies, honoraria, stock ownership or options, expert testimony, grants or patents received or pending, or royalties.

No writing assistance was utilized in the production of this manuscript.

**Table:** 

内容提要
全基因组测序
■ 全基因组测序是对整个基因组的最全面的测序。
■ 全基因组测序是用于癌症基因组深度测序的主要技术。
全外显子组测序
■ 全外显子组测序可对基因组中的编码外显子进行测序。
■ 全外显子组测序更适用于靶标突变的发现。
RNA测序
■ RNA测序产生了完整转录组的全部图谱。
靶向测序
■ 仅对感兴趣的基因组区域进行测序，以期达到节约时间和成本效益的分析。
在既往已知靶标基因中下一代测序的其它发现
■ Marchetti等发现了外显子19的复杂突变和缺失亚群，这可能是携带外显子19缺失的非小细胞肺癌患者间所观察到的反应不同的原因。Su等发现采用EGFR 酪氨酸激酶抑制剂使T790M的发生率从治疗前的31.5%增加至治疗后的83.3%。
■ 复杂的*EML4-ALK*重排可被下一代测序发现，而传统的FISH分析不能检测出这些重排。患者对克唑替尼的反应较好。而且，一个新的扩增融合基因（ *PTPN3-ALK*）已在H2228细胞中成功发现。
■ 在*KRAS*突变样本中发现NF-kB、ERK1/2和AKT通路被激活。
在探索性研究中其他肺癌突变的鉴定
■ 在3项研究中发现肺腺癌（adenocarcinoma, ADC）患者存在*KIF5B*的转换和*RET*基因融合。这一融合可能是潜在的分子靶标。
■ Imielinski等在肺ADC肿瘤/正常DNA配对的研究中绘制出标志物。该研究有助于肺ADC基因组变异的完成和表征。细胞外突变的*ERBB2*的抑制剂可能是有前景的治疗选择。
癌症基因组研究的全局协调
■ 国际癌症基因组协会举国际之力调查癌症的全球图谱并全面识别50种癌症类型的基因组异常。
■ 癌症基因组图谱通过下一代测序技术为超过20种常见癌症的分子特征提供了更全面的认识。
■ 鳞状细胞癌的复杂的基因组变异被发现，包括平均有360个外显子突变、165个基因组重排和323个拷贝数目变异片段。
■ 3个靶标基因为： *ERBBs*、 *FGFRs*和 *JAKs*。
■ Govindan等通过mRNA表达谱发现了30个明显体细胞拷贝数目变异的位点、13个明显突变的基因和4个表达亚型（经典的、基础的、分泌性的和原始的）。
注：本表得到版权所有者 © 2013 Future Medicine Ltd复制许可。
